# PEEP titration in moderate to severe ARDS: plateau versus transpulmonary pressure

**DOI:** 10.1186/s13613-019-0554-3

**Published:** 2019-07-16

**Authors:** Marie Bergez, Nicolas Fritsch, David Tran-Van, Tahar Saghi, Tan Bounkim, Ariane Gentile, Philippe Labadie, Bruno Fontaine, Alexandre Ouattara, Hadrien Rozé

**Affiliations:** 1 Anaesthesia and Intensive Care Unit, Robert Picque Military Teaching Hospital, Villenave d’Ornon, France; 2 Intensive Care Unit, North Bordeaux Aquitaine Clinic, Bordeaux, France; 3 Medical and Surgical Intensive Care, Saint Joseph Saint Luc Teaching Hospital, Lyon, France; 4 Magellan Medico-Surgical Center, South Department of Anaesthesia and Critical Care, CHU Bordeaux, 33000 Bordeaux, France; 5 Biology of Cardiovascular Diseases, INSERM, UMR 1034, Univ. Bordeaux, 33600 Pessac, France

**Keywords:** ARDS, PEEP titration, Monitoring, Transpulmonary pressure

## Abstract

**Background:**

Although lung protection with low tidal volume and limited plateau pressure (*P*_plat_) improves survival in acute respiratory distress syndrome patients (ARDS), the best way to set positive end-expiratory pressure (PEEP) is still debated.

**Methods:**

This study aimed to compare two strategies using individual PEEP based on a maximum *P*_plat_ (28–30 cmH_2_O, the Express group) or on keeping end-expiratory transpulmonary pressure positive (0–5 cmH_2_O, *P*_Lexpi_ group). We estimated alveolar recruitment (Vrec), end-expiratory lung volume and alveolar distension based on elastance-related end-inspiratory transpulmonary pressure (*P*_L,EL_).

**Results:**

Nineteen patients with moderate to severe ARDS (PaO_2_/FiO_2_ < 150 mmHg) were included with a baseline PEEP of 7.0 ± 1.8 cmH_2_O and a PaO_2_/FiO_2_ of 91.2 ± 31.2 mmHg. PEEP and oxygenation increased significantly from baseline with both protocols; PEEP Express group was 14.2 ± 3.6 cmH_2_O versus 16.7 ± 5.9 cmH_2_O in *P*_Lexpi_ group. No patient had the same PEEP with the two protocols. Vrec was higher with the latter protocol (299 [0 to 875] vs. 222 [47 to 483] ml, *p* = 0.049) and correlated with improved oxygenation (*R*^2^ = 0.45, *p* = 0.002). Two and seven patients in the Express and *P*_L,expi_ groups, respectively, had *P*_L,EL_ > 25 cmH_2_O.

**Conclusions:**

There is a great heterogeneity of *P*_Lexpi_ when *P*_plat_ is used to titrate PEEP but with limited risk of over-distension. A PEEP titration for a moderate positive level of *P*_Lexpi_ might slightly improve alveolar recruitment and oxygenation but increases the risk of over-distension in one-third of patients.

## Background

Mechanical ventilation for acute respiratory distress syndrome (ARDS) may lead to ventilation-induced lung injury [1]. A lung protective ventilation strategy, with low tidal volume (*V*_T_), limited plateau pressure and positive end-expiratory pressure (PEEP), aims to improve survival [2, 3]. Different protocols have been proposed to set PEEP in order to avoid alveolar collapse with limited end-inspiratory distension of the lungs [4]. Some of these strategies use a table of PEEP values which depend on inspired fraction of oxygen (FiO_2_), while others are based on individual respiratory mechanics. The Express protocol, developed by Mercat et al., consists of attaining airway plateau pressure (*P*_plat_) up to 28–30 cmH_2_O with a fixed *V*_T_ of 6 ml kg^−1^ predicted body weight [5]. These authors reported a significant reduction in morbidity but not mortality. Because airway pressure is an oversimplified surrogate for lung stress in patients with abnormal chest wall elastance, it could be relevant to assess lung distending pressure estimated from transpulmonary pressure (*P*_L_). This later, in static airway conditions, can be estimated by measuring pleural via esophageal pressure [6]. This estimation can be affected by elastic recoil of the balloon, of the esophagus, esophageal muscular tone and pressures transmitted from the heart beat and mediastinum [7]. The relationship between esophageal and pleural pressure, and its measurement in ARDS patients with an important anteroposterior gradient in the supine position, requires the acceptance of several assumptions [8]. However, a recent study directly measured pleural pressure in pigs and human cadavers and found that esophageal pressure accurately reflects pleural pressure close to the balloon, corresponding to dependent lung regions to mid-chest [7]. Thus, collapse and trauma from recurrent alveolar collapse and re-opening can be related to end-expiratory *P*_L_. [9, 10] Elastance-derived calculation of relative end-inspiratory *P*_L_ (*P*_L,EL_) is close to direct measurement of pleural transpulmonary end-inspiratory pressure in the non-dependent lung region and might therefore give some information about alveolar distension in the non-dependent lung, more at risk for over-distension [11]. Different protocols have been proposed for setting PEEP according to *P*_L_. The EPVent 1 and 2 trials titrated PEEP by measuring pleural pressure to achieve a positive end-expiratory transpulmonary pressure (*P*_Lexpi_) between 0 to 10 cmH_2_O according to a sliding scale based on FiO_2_ [12, 13] Grasso et al. used *P*_L,EL_ to increase PEEP in severe ARDS until *P*_L,EL_ = 25 cmH_2_O [14]. Some patients improved their oxygenation significantly and avoided extracorporeal membrane oxygenation.

The aim of this study was to compare estimated alveolar recruitment (Vrec) with end-expiratory lung volume (EELV) measurement, and alveolar distension with measurement of *P*_L,EL_, during individual PEEP titration using two different targets: *P*_plat_ (28–30 cmH_2_O) or positive *P*_Lexpi_ (0–5 cmH_2_O).

## Methods

### Study design and participants

This multicenter, prospective crossover physiological study was conducted in severe ARDS patients admitted to three French intensive care units in Bordeaux (Robert Picqué Military Teaching Hospital; North Bordeaux Aquitaine Clinic; Thoracic Intensive Care Unit, Bordeaux University Hospital) between 2016 and 2017. All patients had recent (within a week) bilateral opacities not fully explained by cardiac failure or fluid overload with moderate to severe hypoxemia defined by their PaO_2_/FiO_2_ below 150 with 5–8 cmH_2_O of PEEP and required volume-controlled mechanical ventilation [15]. The ventilator used was a carescape R860 (General Electrics, Madison WI, USA).

Exclusion criteria included esophageal disease, pulmonary leakage (major bronchopleural fistula, pneumothorax), severe coagulopathy, solid organ transplantation (hepatic, pulmonary) and refusal to participate.

### Experimental protocol

Sedation was achieved with a midazolam-sufentanil infusion to obtain a bispectral index between 40 and 60. Subjects recieved cisatracurium to obtain myorelaxation, monitoring was Train Of Four of ulnar, 1 or 2 twitches out of 4 was considered appropriate. All subjects were placed in a 30° head up position. A validated nasogastric tube with an esophageal balloon-catheter (Nutrivent™; Sidam, Modena, Italy) was inserted to estimate pleural pressure [16]. The balloon was filled with 4 ml of air. The correct position of the Nutrivent tube was confirmed by an end-expiratory occlusion maneuver with four chest compressions and four ΔPes/ΔPaw ratio measurements as described previously and with thoracic radiography (radio-opaque markers) [6, 17]. At baseline, patients were ventilated with a *V*_T_ of 6 ml kg^−1^ predicted body weight and a PEEP between 5–8 cmH_2_O (PEEP_baseline_). Twenty minutes later, PEEP was titrated according to the Express or *P*_Lexpi_ protocols in a randomized order. For the Express protocol, PEEP was titrated in order to obtain a *P*_plat_ between 28–30 cmH_2_O. For the *P*_Lexpi_ protocol, PEEP was titrated in order to obtain a *P*_Lexpi_ between 0–5 cmH_2_O. PEEP level according to each protocol was maintained for 20 min before recording all respiratory parameters and blood withdrawal for blood gas analysis. PEEP was returned to 0 cmH_2_O between each protocol during less than 30 s. For hemodynamic assessment, respiratory variation of the arterial pulse pressure and the response to a passive leg raising test were used before PEEP titration, with an echocardiography. If positive, a fluid challenge was performed to avoid hypovolemia. After PEEP titration, echocardiography was done to assess right heart function and the occurrence of septal dyskinesia.

### Measurement of variables

End-inspiratory and -expiratory airway and esophageal pressures were measured during a 5 s pause of the ventilator; *V*_T_ were monitored continuously. EELV was measured using the nitrogen washin/washout technique; FiO_2_ variation was 10% and the average of washin EELV and washout EELV for each PEEP levels was recorded.

Variables were calculated using the following equations:

- Absolute inspiratory transpulmonary pressure (*P*_L,es_) = *P*_plat_ − end-inspiratory esophageal pressure;
- Elastance-related transpulmonary pressure (*P*_L,EL_) = *P*_plat_ × (lung elastance/respiratory system elastance) [18];
- *P*_Lexpi_ = total PEEP − end-expiratory esophageal pressure;
- Airway driving pressure (*DP*_aw_) = *P*_plat_ − total PEEP;
- Transpulmonary driving pressure (*DP*_L_) = *DP*_aw_ − (end-inspiratory − end-expiratory esophageal pressure);
- Elastance-related driving pressure = *DP*_aw_ × (lung elastance/respiratory system elastance).
- Respiratory system elastance = (*P*_plat_ − total PEEP)/*V*_T_;
- Lung elastance = *DP*_L_/*V*_T_;
- Respiratory system elastance = Lung elastance + Chest wall elastance.

The following were also measured:

- Estimated recruitment volume (Vrec in ml) = (EELV at high PEEP − EELV at low PEEP) − ((*V*_T_/(*P*_plat_ − low PEEP) × (high PEEP − low PEEP)). [19]

### Statistical analysis

No statistical power calculation was conducted prior to the study; the sample size was based on our previous studies with this design of physiological crossover study with pairing. Data are expressed, respectively, as mean ± standard deviation (SD) and median [interquartile range] for variables normally and non-normally distributed. The outliers were evaluated, but no action was necessary. The categorical data were expressed as numbers (percentage of patients). Comparison of variables between three settings was performed by using one-way repeated measures analysis of variance (ANOVA) followed by post hoc Tukey’s test for multiple comparisons. Comparison between categorical variables was performed using the Chi-squared test. Correlations used Spearman’s test. All statistical tests were two-tailed, and a *p* value of less than 0.05 was considered significant. All statistical analysis was performed using NCSS2007 software (Statistical Solutions Ltd, Cork, Ireland) and Prism 6 (GraphPad Software, La Jolla, CA, USA).

## Results

Nineteen patients were included; they were all enrolled in the study less than 48 h after intubation. The baseline characteristics of these patients are summarized in Table 1, and there were no missing data.

**Table 1:** Baseline characteristics of the patients (*n* = 19)

Characteristic	
Male, *n* (%)	13 (68.4)
Age (years)	72 ± 10
Body mass index (kg/m^2^)	28 ± 6
SAPS II score	65 ± 15
Etiology of ARDS, *n* (%)	
Pneumonia/aspiration	16 (84.2)
Sepsis	2 (10.5)
Pancreatitis	1 (5.3)
Organ failure at baseline (SOFA), *n* (%)	
Hemodynamic	18 (94.7)
Renal	9 (47.4)
Hepatic	0 (0)
Hematological	2 (10.5)
Arterial blood gas	
PaO_2_/FiO_2_ ratio	92 ± 31
FiO_2_ (%)	80 ± 21
pH	7.31 ± 0.11
PaCO_2_ (mmHg)	45 ± 10
HCO_3-_ (mmol/l)	22.4 ± 4.0
Base excess	− 3.4 ± 5.1
Lactates (mmol/l)	1.6 ± 1.0
Hemodynamic variables	
Heart rate (beats/min)	99 ± 27
Systolic arterial pressure (mmHg)	127 ± 23
Diastolic arterial pressure (mmHg)	59 ± 12
Mean arterial pressure (mmHg)	82 ± 13
Respiratory mechanics	
Minute ventilation (L/min)	9.6 ± 1.6
Tidal volume (ml/kg PBW)	6.1 ± 0.4
EELV (ml)	1319 ± 626
Aspect of ARDS, *n* (%)	
Patchy	6 (31.6)
Diffuse	10 (52.6)
Focal	3 (15.8)
Mortality at Hospital discharge	10/19 (53%)

Results are expressed as number (%), or mean ± standard deviation

SAPS 2: Simplified Acute Physiology Score 2; ARDS: acute respiratory distress syndrome; PBW: predicted body weight. SOFA: sepsis-related organ failure assessment; EELV: end-expiratory lung volume (ml)

### PEEP levels, oxygenation and alveolar recruitment

**Fig. 1:**
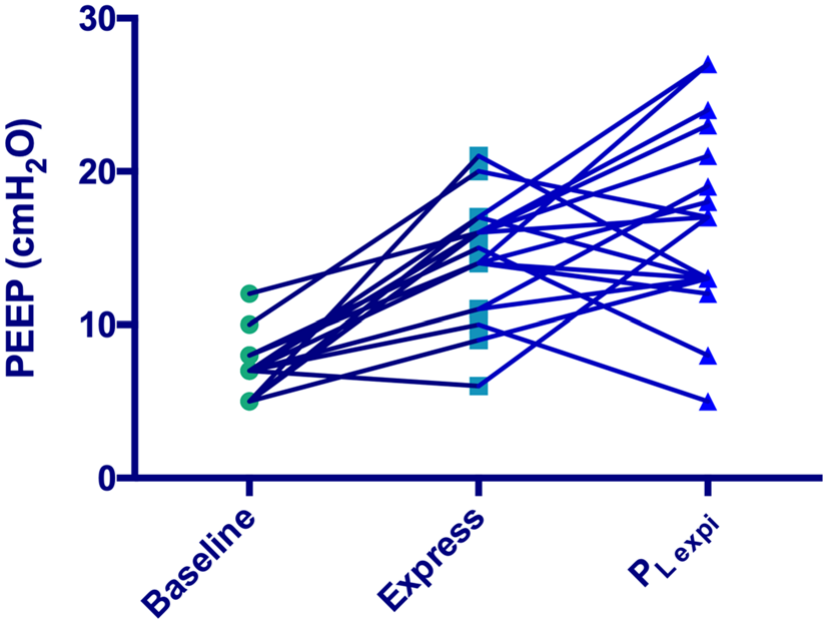
Individual PEEP levels according to the Express or *P*_Lexpi_ protocol. PEEP increased from baseline but is individually different for almost all patients with each protocol Express or *P*_Lexpi_

Respiratory mechanics according to each PEEP setting are summarized in Table 2. In comparison with PEEP_baseline_, the Express and *P*_Lexpi_ protocols significantly increased PEEP and *P*_plat_ without any change in driving pressure (Table 2). These changes were associated with a significant improvement in oxygenation. Median PEEP value was not significantly different between the Express and *P*_Lexpi_ protocols. However, analysis of individual PEEP data according to each protocol shows that no patient had the same PEEP (Fig. 1) with a median of absolute difference of 5.0 [4.0–8.0] cmH_2_O and 13 patients had higher PEEP with *P*_Lexpi_ protocol.

**Table 2:** Measurements of respiratory function and hemodynamics (*n* = 19)

Protocols	PEEP_baseline_	Express protocol	*P*_Lexpi_	*p* value
PEEP (cmH_2_O)	7.0 ± 1.8	14.2 ± 3.6*	16.7 (5.9)*	< 0.0001
*P*_plat_ (cmH_2_O)	20.8 ± 4.0	28.8 ± 2.0 *	33.9 ± 10.6*	< 0.0001
*P*_L,es_ (cmH_2_O)	7.0 ± 5.9	11.9 ± 6.2*	15.5 ± 8.5*	0.0013
*P*_L,EL_ (cmH_2_O)	15.3 ± 4.9)	20.5 ± 4.7*	24.3 ± 11.4*	0.0025
*P*_Lexpi_ (cmH_2_O)	− 2.6 ± 5.2	1.4 ± 5.1*	3.3 ± 1.6*	< 0.0001
EELV (ml)	1546 ± 634	2067 ± 924*	2287 ± 945*	0.001
*DP*_aw_ (cmH_2_O)	13.0 ± 3.9	14.2 ± 5.0	16.4 ± 7.8	0.17
*DP*_L_ (cmH_2_O)	9.9 ± 4.4	10.6 ± 5.6	12.3 ± 8.3	0.20
*DP*_L,EL_ (cmH_2_O)	7.5 ± 4.3	8.1 ± 5.6	9.5 ± 8.1	0.30
Crs (ml/cmH_2_O)	33.3 ± 15.8	30.0 ± 10.7	28.3 ± 13.2	0.17
*E*_cw_ (cmH_2_O/l)	8.7 ± 2.7	9.6 ± 3.4*	10.9 ± 4.3*	0.03
*E*_L_ (cmH_2_O/l)	26.0 ± 11.9	28.0 ± 15.9	33.2 ± 25.1	0.25
FiO_2_ (%)	80.0 ± 21.1	80.6 ± 21.2	81.1 ± 21.6	0.46
PaO_2_/FiO_2_	91.2 ± 31.2	134.0 ± 67.2*	152.7 ± 80.1*	0.01
pH	7.31 ± 0.11	7.30 ± 0.11	7.31 ± 0.12	0.08
PaCO_2_ (mmHg)	45.2 ± 10.4	46.5 ± 9.6	45.3 ± 11.0	0.26
MAP (mmHg)	82.0 ± 13.4	74.7 ± 12.9	75.7 ± 12.0	0.06
Heart rate (beats/min)	99 ± 27	102 ± 26	107 ± 28	0.19
Lactates (mmol/l)	1.6 ± 0.9	1.5 ± 0.8	1.5 ± 0.8	0.27

Results are expressed as mean ± standard deviation

*P*_plat_: plateau pressure; *P*_Lexpi_: end-expiratory transpulmonary pressure; *DP*_L_: transpulmonary driving pressure; *P*_L,EL_: relative end-expiratory pressure; *P*_L,es_: absolute inspiratory transpulmonary pressure; *DP*_aw_: airway driving pressure; *DP*_L_: transpulmonary driving pressure, *DP*_L,EL_: transpulmonary elastance-related driving pressure; *E*_L_: lung elastance; EELV: end-expiratory lung volume; *E*_cw_: elastance chest wall; Crs: compliance respiratory system; MAP: mean arterial pressure. *p* value refers to repeated measures ANOVA. *p < 0.05 of Express and *P*_Lexpi_ groups versus baseline group. ^§^*p* < 0.05 of Express versus *P*_Lexpi_ groups

EELV variation from baseline was significantly higher with *P*_Lexpi_ protocol 60 (58) % vs 36 (28) % with Express protocol, *p* = 0.025. Estimated Vrec was significantly higher with the *P*_Lexpi_ protocol than with Express, 298 [0 to 845] vs 222 [47 to 483] ml, respectively. Vrec and PaO_2_/FiO_2_ ratio changes were significantly correlated with the *P*_Lexpi_ protocol (*R*^2^ = 0.45, *p* = 0.002). Arterial blood gases were not significantly different between the Express and *P*_Lexpi_ protocols.

## Expiratory transpulmonary pressure

**Fig. 2:**
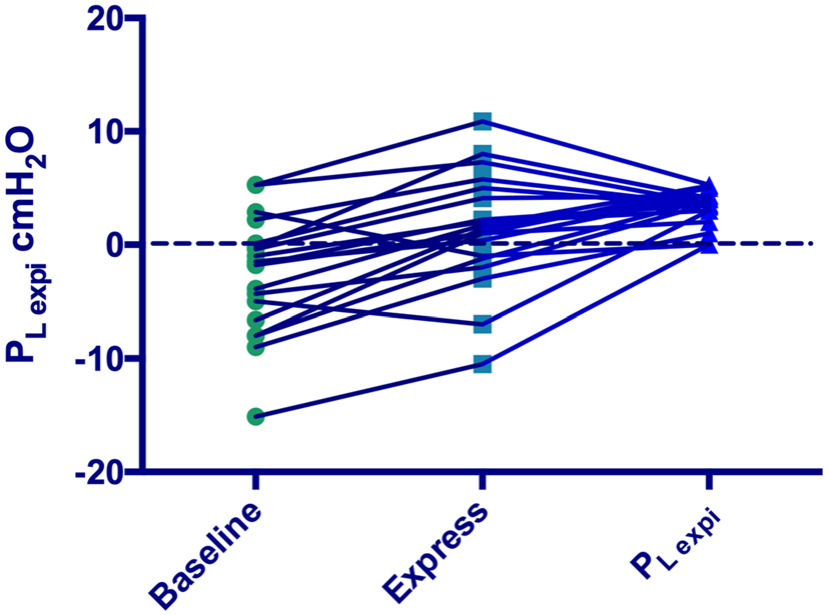
Individual *P*_Lexpi_ levels according to baseline, Express and *P*_Lexpi_ protocols. *P*_Lexpi_ = positive end-expiratory transpulmonary pressure. Dash line represents the limit of 0 cmH_2_O; more patients had negative *P*_Lexpi_ with the Express protocol

*P*_Lexpi_ increased with both protocols (Table 2, Fig. 2).

With the Express protocol, six patients had negative *P*_Lexpi_ − 2.5 [− 7.8 to − 1.15] cmH_2_O with a median PEEP of 14.0 [9.7 to 14.5] cmH_2_O. These six patients had a significant lower respiratory system compliance (25.2 [15.8 to 29.0] vs. 30.7 [25.6 to 39.2] ml/cmH_2_O, *p* = 0.027), and a significant lower EELV (1240 [835 to 2118] vs. 2019 [1700 to 3045] ml, *p* = 0.040). When these patients received the *P*_Lexpi_ protocol, median PEEP increased to 24.0 [18.0 to 26.5] cmH_2_O, *P*_plat_ was 40.2 [33.1 to 51.0] cmH_2_O, and PaO_2_/FiO_2_ increased by +31 [11 to 206] % (*p* = 0.062).

### Inspiratory transpulmonary pressure

**Fig. 3:**
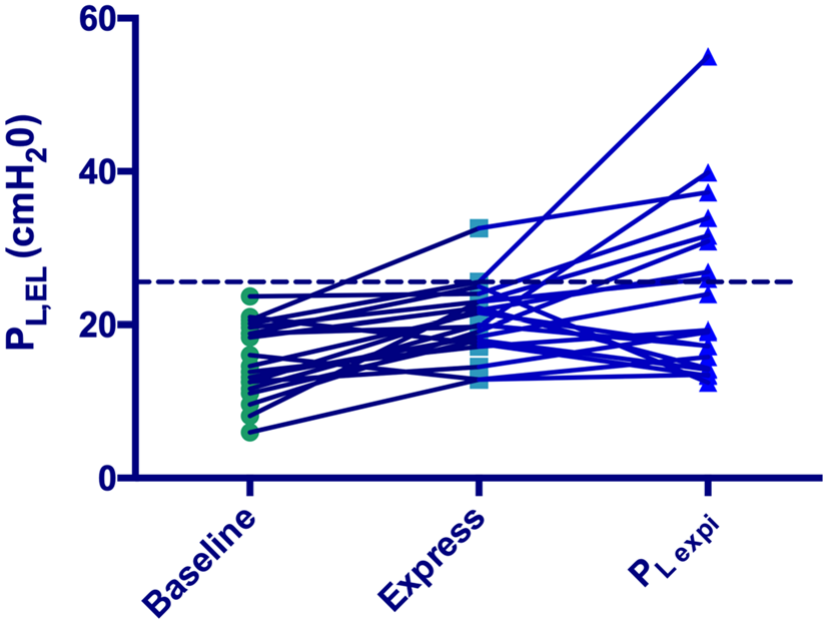
Individual *P*_L,EL_ with baseline, Express and *P*_Lexpi_ protocols. *P*_L,EL_ = elastance-derived calculation of relative end-inspiratory transpulmonary pressure. Dash line represents the limit of 25 cmH_2_O; more patients had *P*_L,EL_ above 25 cmH_2_O with the *P*_Lexpi_ protocol

With the Express protocol, mean *P*_L,EL_ was 20 cmH_2_O, and *P*_L,es_ was significantly lower than *P*_L,EL_ with a mean of difference of 8.5 cmH_2_O (*p* < 0.0001) (Table 2). *P*_L,EL_ increased from baseline with the Express protocol (Table 2), one patient was > 25 cmH_2_O, and the rest were below (Fig. 3), but 2 patients had *P*_L,es_ > 20 cmH_2_O.

With the *P*_Lexpi_ protocol, mean *P*_L,EL_ was 24 cmH_2_O, and *P*_L,es_ was significantly lower than *P*_L,EL_ with a mean of difference of 8.7 cmH_2_O (*p* < 0.0001) (Table 2). *P*_L,EL_ increased from baseline with the *P*_Lexpi_ protocol (Table 2), seven patients had *P*_L,EL_ > 25 cmH_2_O (Fig. 3), and four of them had *P*_L,es_ > 20 cmH_2_O.

With the *P*_Lexpi_ protocol, patients with *P*_L,EL_ > 25 cmH_2_O had pH of 7.25 [7.17 to 7.42] with significantly lower respiratory system compliance, higher DP and PEEP, than those with *P*_L,EL_ ≤ 25 cmH_2_O (17 [15 to 22] ml/cmH_2_O, 23 [18 to 25] cmH_2_O and 21[17 to 27] cmH_2_O, respectively).

Airway, transpulmonary and elastance-related driving pressures were not different between groups (Table 2).

### Elastance

In comparison with baseline, respiratory system elastance and lung elastance were not significantly modified by the increase in PEEP with the Express protocol; only chest wall elastance was statistically different (Table 2). PEEP increase from baseline with the Express protocol was significantly correlated with lung elastance (*R*^2^ = 0.38, *p* = 0.008). Respiratory system and lung elastance were similar between Express and *P*_Lexpi_ protocols.

### Complications

No episodes of pneumothorax were detected by chest X-Ray after each protocol implementation. Hemodynamic assessment was done before PEEP titration, and fluid was given when required. Cardiac frequency and mean arterial pressure remained relatively stable with each PEEP protocol (Table 2). Fifteen patients underwent transthoracic echocardiography (four had no transthoracic echocardiography window), and none of them had right heart failure with septal dyskinesia after PEEP titration.

## Discussion

This study demonstrates heterogeneity of end-expiratory or end-inspiratory transpulmonary pressures during PEEP titration with each method in severe ARDS. PEEP titration with a target of positive *P*_Lexpi_ will suggest individually different levels of PEEP than with Express protocol in a large majority of patients (higher or lower PEEP). These two protocols are not interchangeable, as described previously with two other protocols based on expiratory *P*_L_ [20, 21].

The goal of increasing PEEP from baseline with a specific titration protocol using *P*_L_ is to improve alveolar recruitment and oxygenation with limited hyperinflation and morbi-mortality [4]. ARDS patients have a great variation in lung damage with a gravitational vertical gradient of alveolar injuries determining dependent and non-dependent regions [22]. Pleural pressure also has a vertical gradient in the supine position [23]. Interestingly, *P*_L,EL_ is close to direct measurement of pleural transpulmonary end-inspiratory pressure in the non-dependent lung region and might therefore limit the risk of ventilator-induced lung injury by over-distension [11]. Our main results demonstrate that this risk is probably reduced when PEEP is titrated according to *P*_plat_ (28–30 cmH_2_O) instead of a moderate positive *P*_Lexpi_. (0–5 cmH_2_O).

### Oxygenation and alveolar recruitment, according to *P*_Lexpi_

In our study, when compared to baseline, the Express and *P*_Lexpi_ protocols increased PEEP, Vrec and oxygenation at the same time significantly. These two protocols based on respiratory mechanic proposed high PEEP level which might explain the small differences in recruitment. In the 2 EPVent trials, the difference in the results might come from the comparator group with empirical PEEP-FiO_2_ strategies, resulting in significantly different *P*_Lexpi_ on protocol between groups. *P*_Lexpi_ protocol proposed individually a different level of PEEP than Express protocol. The corresponding increase of PEEP with *P*_Lexpi_ protocol, when compared from baseline, was correlated to oxygenation. Patients with protective ventilation who respond to increased PEEP by improved oxygenation might have a lower risk of death [3]. The *P*_Lexpi_ protocol had a better alveolar recruitment than Express protocol with a median *P*_Lexpi_ of 3.6 cmH_2_O. Indeed, increasing PEEP in order to have a positive *P*_Lexpi_ of around 4.5 cmH_2_O can significantly reduce the risk of atelectasis in the dependent lung, whereas a *P*_Lexpi_ of 0 cmH_2_O might not be enough [23].

With the Express protocol, six patients had negative *P*_Lexpi_, which meant that esophageal pressure was greater than airway pressure. This may occur when pressures in the thorax and abdomen are pathologically elevated [24]. These patients had lower respiratory system compliance and EELV. With the *P*_Lexpi_ protocol, these patients received higher levels of PEEP and a trend toward increased oxygenation. This protocol might improve alveolar recruitment and oxygenation, but over-distension has to be controlled at the same time [4].

### Over-distension, according to *P*_L,EL_

In order to limit over-distension, *P*_plat_ should remain < 32 cmH_2_O and *DP*_aw_ < 15 cmH_2_O [2, 25]. *P*_L,EL_ can also help to limit over-distension in animal study based on CT scan [26], and a limit of 25 cmH_2_O has been proposed in ARDS patients [13]. We found that *DP*_aw_ remained unchanged and within a safer range below 15 cmH_2_O with all protocols [25]. *DP*_aw_ and *DP*_L_ were highly correlated, and the mean difference was similar to previous data (approximately 4 cmH_2_O) [27]. The EPVent trial 2 proposed stopping the PEEP increase if *P*_L,es_ was > 20 cmH_2_O [13]. With the *P*_Lexpi_ protocol, four patients had *P*_L,es_ > 20 cmH_2_O, but seven patients had *P*_L,EL_ > 25 cmH_2_O. This can be explained by a significant difference between these two assessments of end-inspiratory transpulmonary pressure as described previously [9, 23]. With the Express protocol, PEEP was titrated on the basis of *P*_plat_, with the expectation that it closely approximated *P*_L_. In patients with normal chest wall elastance, *P*_plat_ is a reasonable surrogate for *P*_L_ and with Express protocol, we found a median *P*_L,EL_ of 20 cmH_2_O which is lower than the target/limit of 25 cmH_2_O proposed by Grasso et al. [11]; this limit was crossed by only one patient in our study.

With the *P*_Lexpi_ protocol, Vrec was slightly higher, but some patients had *P*_L,EL_ > 25 cmH_2_O; these patients had lower respiratory system compliance with higher DP and respiratory acidosis. Thus, for some patients having both moderate positive *P*_Lexpi_ and limited *P*_L,EL_ is not possible, and moderate positive *P*_Lexpi_ between 0–5 cmH_2_O does not prevent *P*_L,EL_ being > 25 cmH_2_O. In these patients, *P*_L,EL_ may need to be reduced and *V*_T_ might be decreased below 6 ml kg^−1^ PBW in order to benefit from PEEP-induced alveolar recruitment with less distension and DP. The use of the prone position could also be interesting [28], but assessment of pleural pressure by esophageal pressure in prone position requires further investigations.

### Elastance

Lung elastance in our patients was similar to previous studies [13, 26]. When chest wall elastance is high, *P*_plat_ may be much higher than *P*_L_. Indeed, a substantial portion of *P*_plat_ is dissipated in distending the chest wall. As the chest wall becomes stiffer, the proportion of *P*_plat_ that distends the lung (*P*_L_) decreases progressively [29]. Chest wall elastance was within the same range as other data in ARDS patients [12, 13]. In the study of Grasso et al., patients improved by PEEP had a higher chest wall elastance with a mean of 17 cmH_2_O/l. Chest wall elastance can be elevated in patients with acute respiratory failure for various reasons [30]. Increases in chest wall elastance can occur as a result of intra-abdominal hypertension, pleural effusion, massive ascites, thoracic trauma and edema of the intra-thoracic and intra-abdominal tissues as a result of fluid resuscitation [7]. We found a significant increase of *E*_cw_ with *P*_lexpi_ protocol. This has already been described by Mezidi et al. and might be explained by an upward shift of the chest wall pressure–volume curve with more EELV [31].

### Severity and complications

In our cohort, patients were severely ill with high Simplified Acute Physiology Scores II (SAPS II) and PaO_2_/FiO_2_ ratio at baseline was lower than in most studies including the EPVent trials [12, 13], Express study [5] and ARMA study [2]. Titration of PEEP was achieved with the *P*_Lexpi_ and Express protocols in all patients without hemodynamic impairment.

### Limits and perspectives

There are several limitations. First of all, it is a small physiological study of moderate to severe ARDS patients with the risk of underpowered statistical analysis. We assessed alveolar recruitment and the risk of over-distension only with bedside respiratory mechanics and not with a CT scan that is the gold standard. This study did not assess alveolar inflammation, and we did not assess any outcome according to a specific PEEP titration protocol. The majority of the patients had a direct insult of the lung with pneumoniae or aspiration so it was not possible to compare respiratory mechanic between pulmonary and extrapulmonary ARDS [32]. All patients had a risk of derecruitment with a short ventilation with no PEEP between each protocol. Some of these limits need further investigations.

## Conclusions

Our study demonstrates that, in comparison with a protocol using *P*_plat_ to titrate PEEP, a positive level of *P*_Lexpi_ with PEEP might slightly improve alveolar recruitment and oxygenation but also increases *P*_L,EL_ above 25 cmH_2_O and the risk of over-distension in the dependent lung in one-third of patients.

## Data Availability

If requested.

## Prior presentation

This study was presented in ESICM Lives Forum 2018 in Madrid as best abstract.
